# Healthcare seeking patterns for TB symptoms: Findings from the first national TB prevalence survey of South Africa, 2017–2019

**DOI:** 10.1371/journal.pone.0282125

**Published:** 2023-03-15

**Authors:** Sizulu Moyo, Farzana Ismail, Nkateko Mkhondo, Martie van der Walt, Sicelo S. Dlamini, Thuli Mthiyane, Inbarani Naidoo, Khangelani Zuma, Marina Tadolini, Irwin Law, Lindiwe Mvusi

**Affiliations:** 1 Human Sciences Research Council, Cape Town, South Africa; 2 School of Public Health and Family, University of Cape Town, Cape Town, South Africa; 3 National Institute for Communicable Diseases Division of the National Health Laboratory Services, Johannesburg, South Africa; 4 Department of Medical Microbiology, University of Pretoria, Pretoria, South Africa; 5 Tuberculosis Programme, World Health Organization, Pretoria, South Africa; 6 South African Medical Research Council, Pretoria, South Africa; 7 National Department of Health, Pretoria, South Africa; 8 Infectious Diseases Unit, IRCCS Azienda Ospedaliero-Universitaria di Bologna, Bologna, Italy; 9 Department of Medical and Surgical Sciences, Alma Mater Studiorum University of Bologna, Bologna, Italy; 10 Global Tuberculosis Programme, World Health Organization, Geneva, Switzerland; Stellenbosch University, SOUTH AFRICA

## Abstract

**Background:**

Although tuberculosis (TB) symptoms have limited sensitivity they remain an important entry point into the TB care cascade.

**Objectives:**

To investigate self-reported healthcare seeking for TB symptoms in participants in a community-based survey.

**Methods:**

We compared reasons for not seeking care in participants reporting ≥1 of four TB screening symptoms (cough, weight loss, night sweats, fever) in the first South African national TB prevalence survey (2017–2019). We used logistic regression analyses to identify sociodemographic and clinical characteristics associated with healthcare seeking.

**Results:**

5,168/35,191 (14.7%) survey participants reported TB symptoms and 3,442/5168 had not sought healthcare. 2,064/3,442(60.0%) participants intended to seek care, 912 (26.5%) regarded symptoms as benign, 399 (11.6%) reported access barriers(distance and cost), 36 (1.0%) took other medications and 20(0.6%) reported health system barriers. Of the 57/98 symptomatic participants diagnosed with bacteriologically confirmed TB who had not sought care: 38(66.7%) intended to do so, 8(14.0%) regarded symptoms as benign, and 6(10.5%) reported access barriers. Among these 98, those with unknown HIV status(OR 0.16 95% CI 0.03–0.82), p = 0.03 and those who smoked tobacco products(OR 0.39, 95% CI 0.17–0.89, p = 0.03) were significantly less likely to seek care.

**Conclusions:**

People with TB symptoms delayed seeking healthcare, many regarded symptoms as benign while others faced access barriers. Those with unknown HIV status were significantly less likely to seek care. Strengthening community-based TB awareness and screening programmes together with self-screening models could increase awareness of the significance of TB symptoms and contribute to improving healthcare seeking and enable many people with TB to enter the TB care cascade.

## Background

Over the past two decades many countries have made significant commitments to end tuberculosis (TB) through the adoption of the World Health Organization (WHO) End TB Strategy, and the United Nations (UN) Sustainable Development Goals (SDGs) [1, 2] among other national commitments. It is against this background that significant progress has been made in TB diagnostics and treatment [3–5]. However, in high TB burden settings many people with TB remain undetected, undiagnosed and untreated [6, 7]. Additionally, in the past three years, COVID-19 regressed the work and gains of health programmes including TB programmes [6, 8]. COVID-19 related disruptions drastically reduced TB testing and notifications and disrupted the supply and provision of TB medication and clinical care for people with TB resulting in an increase in global TB mortality in 2020 [6, 8].

Recovering, improving, and sustaining progress to eliminate TB requires interventions throughout the TB care cascade. This includes addressing barriers to entry into the care cascade in combination with availability and accessibility of rapid and highly sensitive diagnostic tools, and short treatment regimens with minimal adverse effects. Although the four common TB symptoms alone have poor sensitivity in screening for TB [9],they remain a significant entry point into the TB care cascade for many people who have TB. In this paper we analyze data on self-reported healthcare seeking in individuals with TB symptoms who participated in the first national TB prevalence survey undertaken in South Africa in 2017–2019. As South Africa and other countries seek to find and treat more people with TB, such analyses can inform efforts to improve and support screening, by identifying the main barriers preventing healthcare seeking among those with symptoms suggestive of TB and provide evidence for interventions to offer them testing and treatment services more efficiently.

## Methods

We used data from the first South African national TB prevalence survey undertaken in 2017–2019 [10]. The detailed survey methodology has been described elsewhere [10]. In summary, the survey enrolled eligible participants aged 15 years and older in 110 clusters sampled by probability proportional to size across all the 9 provinces of the country. Those enrolled were screened for TB using four TB screening symptoms [9]: persistent cough of any duration (we also asked about the duration of the cough), unexplained weight loss, unexplained fever for ≥2 weeks and drenching night sweats for ≥2 weeks, and by digital chest X-ray (CXR). Symptoms were elicited by trained interviewers using a structured questionnaire. The questionnaire also collected data on collected data on selected behaviours, health status and reasons for not seeking care for TB symptoms among those reporting symptoms. Participants could select from the following reasons a) Distance- facility is far from where I live, b) Money- I had no money for transport to the health centre, c) Importance/relevance of symptoms- I did not consider it to be important, d) Still planning to seek care, and e) Other reasons-(open ended to capture all other reasons). Participants could select and report multiple reasons. Optional data on self-reported HIV status was also collected. Those who screened positive (symptomatic and/or CXR changes suggestive of active TB) submitted two sputum samples for testing by Xpert MTB/RIF Ultra assay (Cepheid, Sunnyvale, CA, USA) and liquid culture (Bactec MGIT 960 Becton Dickinson, Franklin Lakes, NJ, USA) and dried blood spot(DBS) samples for HIV testing if they consented to HIV testing. Sputum samples were processed at the Centre for Tuberculosis at the National Institute for Communicable Diseases (NICD). HIV testing was performed by the Centre for HIV and STIs (CHIVSTI) at the NICD. Participants who had *Mycobacterium tuberculosis* complex culture-positive sputum samples were considered positive for bacteriologically confirmed pulmonary TB. In the absence of a positive culture (i.e., negative, contaminated, or not done), participants were considered positive for bacteriologically confirmed pulmonary TB in the presence of an Xpert MTB/RIF Ultra-positive sample, together with an abnormal CXR suggestive of active TB (determined by central panel CXR readers), and no history of past TB. The final HIV status was based on the DBS result where available, and on self-reported status otherwise. HIV status was unknown where participants declined to disclose their status and refused DBS testing.

### Data analysis

We analysed data on self-reported healthcare seeking for survey participants who reported one or more of the four screening symptoms. We used STATA v15.0 and summarized the data using frequencies, proportions, medians and IQRs as appropriate, and used the Pearson’s Chi Square test to compare categorical variables, with p <0.05 indicating statistical significance. We assessed factors associated with seeking care using bivariate and multivariate logistic regression analysis. Variables that were significant in bivariate analyses were included in a multivariate regression analysis with variables simultaneously adjusted for each other. We report odds ratios (ORs) and adjusted odds ratios (aORs) with 95% confidence intervals (CIs) and p values, and p <0.05 indicates statistical significance.

We initially grouped the data on reasons for not seeking healthcare into four categories as i) those who were planning to seek care (Still planning to seek care), ii) those facing access barriers (cost and distance;-Distance- facility is far from where I live, and Money- I had no money for transport to the health centre), iii) those who regarded their symptoms as benign (Importance/relevance of symptoms- I did not consider it to be important) and iv) Other reasons. We further examined the Other reasons category and allocated these to categories i) to iii) above as appropriate and also created two additional categories that captured health facility factors (e.g., the clinic congestion and poor staff attitudes) and use of other medication (use of over-the-counter medication, or traditional medication).

### Ethics

The survey protocol was approved by the South African Medical Research Council Research Ethics Committee (EC001 2/2012). Written informed consent or assent and parent or guardian consent for participants younger than 18 years old was obtained before enrolment into the survey.

## Results

Of the 35,191 participants enrolled in the survey 5,168(14.7%), median age of 47 years (IQR 32–61), and mostly women (3,064, (59.3%)), reported at least one of the four TB symptoms. Overall, 2,968 participants reported one symptom only and this was most frequently cough (n = 1,400 (47.2%)) followed by night sweats (n = 660 (22.2%)), weight loss (n = 641 (21.6%)), and fever(n = 267 (9.0%)). 3,442 /5,168 (66.6%) participants reporting symptoms had not sought care for them. The majority, 2,064 (60.0%) reported that they intended to seek care, 912(26.5%) regarded their symptoms as benign, and 399 (11.6%) reported access barriers to healthcare seeking (distance—healthcare facilities far from where they lived and cost—unable to afford travel costs). There were 36 (1.0%) participants who reported that they were taking other medications for their symptoms, while 20(0.6%) people did not seek care because of health system related factors (facility congestion, poor staff attitudes), and 4(0.1%) who were afraid of a possible diagnosis of TB. In a further 7 participants no reasons were given (S1 Table).

There was no statistically significant difference in the proportions of those who reported that they intended to seek care by sex, diabetes, and past history of TB, (Table 1). More participants aged 25–49 years old (62.6% 25-49years, 57.5% 15-24years, 58.1% ≥ 50years), living in urban areas(64.9% urban, 55.2% rural, p = 0.000), those with Grade 1–12 education (53.9% no education, 61.4% Grade 1–12, 56.6% tertiary level, p = 0.003), those with known HIV status (62.9% HIV+ve, 61.7% HIV-ve, 52.3% HIV status unknown, p< = 0.000), those who reported smoking tobacco products (62.7% smokers, 58.3% nonsmokers, p = 0.01), and those who reported no alcohol intake(not taking alcohol 61.6%, 57.8 taking alcohol, p = 0.03) intended to seek care for their symptoms (Table 1). Among the 558 symptomatic participants aged 15–24 years old who had not sought care, 31.7% regarded their symptoms as benign and this proportion was significantly greater than in the older age groups (26.4% among those 25–49 years old and 24.5% among those ≥ 50 years old, p = 0.01). A significantly greater proportion of those with tertiary education (31.9% tertiary level, 27.3% Grade 1–12 and 21.3% no education, p = 0.01), those with unknown HIV status (30.9% HIV status unknown, 23.7% HIV+ve, 25.9% HIV-ve, p = 0.01), those with no history of past TB (27.2% no past history of TB, 21.1% with history of past TB, p = 0.01), and those who consumed alcohol (consume alcohol 30.2%, 23.7% no g alcohol, p = 0.000) regarded their symptoms as benign (Table 1). Access barriers were most frequently reported by females (9.9% males, 12.9% females, p = 0.01), those ≥50 years (15.9% in those ≥50 years old, 9.3% in those 25–49 years old, 7.34% 15–24 years old, p = 0.000), those with no education (23.8% no education, 9.4% Grade 1–12 and 3.5% tertiary, p = 0.000), and those living in rural areas (17.1% rural, 5.8% urban, p = 0.000), those with unknown HIV status (HIV status unknown 15.2%, HIV -ve 10.3%, HIV +ve 12.1%, p = 0.002), those who did not consume alcohol (no alcohol 12.6%, 10.1% taking alcohol, p = 0.02) and those who did not smoke cigarettes (13.1% nonsmokers, 9.1% smokers, p = 0.000) (Table 1).

**Table 1:** Reasons for not seeking care for TB symptoms, N = 3,442.

		Reason for not seeking care			
**Variable**	**Did not seek care n**	**Still planning to seek care n (%)**	**Symptoms regarded as benign n (%)**	**Access barriers n (%)**	**Other reasons**
**Sex**		p = 0.55	p = 0.06	p = 0.01	
Male	1,500	891 (59.4)	422 (28.1)	149 (9.9)	38
Female	1,942	1,173 (60.4)	490 (24.5)	250 (12.9)	29
**Age group (years)**		p = 0.02	p = 0.01	p = 0.001	
15–24	558	321 (57.5)	177 (31.7)	43 (7.7)	34
25–49	1,522	952 (62.6)	402 (26.4)	140 (9.2)	28
≥50	1,362	791 (58.1)	333 (24.5)	216 (15.9)	22
**Locality**		p = 0.000	p = 0.33	p = 0.000	
Urban	1,677	1089 (64.9)	457 (27.3)	98 (5.4)	33
Rural	1,765	975 (55.2)	455 (25.8)	301 (17.1)	76
**Highest education level achieved**		p = 0.002	p = 0.01	p = 0.000	
None	572	308(53.9)	122 (21.3)	136 (23.8)	6
Grade 1–12	2,755	1,692 (61.4)	752 (27.3)	259 (9.4)	52
Tertiary	113	64 (56.6)	36 (31.9)	4 (3.5)	9
Missing	2				
^**#**^ **HIV status**		p = 0.000	p = 0.01	p = 0.002	
HIV positive	574	361 (62.9)	136 (23.7)	70 (12.2)	7
HIV negative	2,164	1,335 (61.7)	560 (25.9)	222 (10.3)	47
HIV status unknown	704	368 (52.3)	216 (30.9)	107 (15.2)	13
**Diabetes (self-report)**		p = 0.66	p = 0.47	P = 0.15	
No	3,124	1,881 (60.2)	832 (26.6)	352 (11.3)	59
Yes	207	118 (57.0)	55 (26.6)	30 (14.5)	4
Don’t know	108	65 (60.2)	23 (21.3)	17 (15.7)	3
Missing	3				
**History of past TB**		p = 0.39	p = 0.01	p = 0.11	
Yes	445	276 (62.0)	94 (21.1)	61 (13.7)	14
No	2,997	1,782 (59.9)	811 (27.2)	331 (11.1)	73
unknown	20				
**Smoke tobacco products**		p = 0.01	p = 0.70	p = 0.000	
No	1,996	1,163 (58.3)	521 (26.1)	268 (13.4)	44
Yes	1435	899 (62.7)	383 (26.7)	130 (9.1)	23
Missing	11				
**Consume alcohol**		p = 0.03	p = 0.000	p = 0.02	
No	2,006	1,236 (61.6)	476 (23.7)	253 (12.6)	41
Yes	1,423	823 (57.8)	430 (30.2))	143 (10.1)	27
Missing	13				

^#^ HIV status was determined by dried blood spot(DBS) test results where this was available and by self-report where DBS was not done. HIV status was unknown where participants declined to disclose status and refused DBS testing

When the analyses excluded participants reporting cough of less than 2 weeks, there was no statistically significant difference in the proportions of those who reported that they intended to seek care by sex, age group, past history of TB, and diabetes (S2 Table). More participants living in urban areas, those with Grade 1–12 education and those who smoked tobacco products intended to seek care, while significantly lower proportions of those with unknown HIV status and those who consumed alcohol intended to seek care. Findings were also similar for those who regarded their symptoms as benign. Significantly more young people 15–24 years old, those with a tertiary level of education those with unknown HIV status, and those who consumed alcohol regarded their symptoms as benign. Women, the elderly, those living in rural areas, those with unknown HIV status, those who did not smoke, and those who did not consume alcohol most frequently reported access barriers. (S2 Table).

Reasons for not seeking healthcare were heterogenous across all of the four screening symptoms, across different combinations of symptoms and number of concurrent symptoms (S3 Table). Nearly a third of those reporting cough only, and a third of those who reported all four symptoms were planning to seek care. When stratified by HIV status, 72.7% of those reporting all four symptoms and were HIV+ve were planning to seek care, compared to 61.0% of those who were HIV-ve and 62.5% of those with unknown HIV status. Among those who reported cough only, 66.7%, 66.5% and 55.6% were planning to seek care for the cough among those living with HIV (LWH), those without HIV and those with unknown HIV status respectively. These proportions were largely unchanged when analysis was restricted to cough of 2 weeks or longer, at 71.5% for those HIV-positive, 60.0% among those HIV-ve and 60.5% among those with unknown status.

### Factors associated with self-reported care seeking for TB symptoms

Overall, older people were more likely to seek care for their symptoms, with those ≥50 years old 3 times (aOR 3.14, 95% CI 2.50–3.96) more likely to seek care compared to those 15–24 years old. Participants living in rural areas were also more likely to seek care than those living in urban areas (aOR 1.71, 95% CI 1.03–1.33), as were those with a history of past TB (aOR 1.61, 95% CI 1.37–1.90), and those with diabetes (aOR 1.48, 95% CI 1.18–1.84) (Table 2). Participants who were HIV-ve (aOR 0.57, 95% CI 0.49–0.67) and those with unknown HIV status (aOR 0.45,95% CI 0.37–0.56) were less likely to seek care for their symptoms as were those who smoked cigarettes (aOR 0.64, 95% CI 0.54–0.74) and those who consumed alcohol (aOR 0.81, 95% CI 0.70–0.94) (Table 2).

**Table 2:** Factors associated with self-reported care seeking for TB symptoms in a community survey, N = 5168.

Characteristic	Number reporting symptoms	Sought care n(%)	OR (95% CI)	p value	aOR (95% CI)	p value
**Sex**						
Male	2,104	604 (28.7)	ref		ref	
Female	3, 064	1,122 (36.6)	1.44 (1.27–1.62)	0.000	1.03 (0.89–1.19)	0.68
**Age group (years)**						
15–24	678	120 (17.7)	ref		ref	
25–49	2,121	599 (28.2)	1.83 (1.47–2.28)	0.000	1.66 (1.32–2.09)	<0.001
≥50	2,369	1007 (42.5)	3.43 (2.78–4.26)	0.000	3.14 (2.50–3.96)	<0.001
**Locality**						
Urban	2,381	704 (29.6)	ref		ref	
Rural	2,787	1,022 (36.7)	1.38 (1.23–1.55)	0.000	1.17 (1.03–1.33)	0.02
**Highest education level achieved**						
None	998	426 (42.69)			ref	
Grade 1–12	4,009	1,254 (31.28)	0.61 (0.53–0.70)	0.000	0.89 (0.75–1.04)	0.15
Tertiary	1,57	44 (28.03)	0.52 (0.36–0.76	0.000	0.98 (0.66–1.46)	0.93
Missing	4					
^**#**^ **HIV status** ^**#**^						
HIV-positive	1,017	443 (43.6)			ref	
HIV-negative	3,156	992 (31.4)	059 (0.51–0.69)	0.000	0.57 (0.49–0.67)	0.000
HIV status unknown	995	291 (29.3)	0.54 (0.45–0.64)	0.000	0.45 (0.37–0.56	0.000
**Diabetes (self-report)**						
No	4,628	1,504 (32.5)	ref		ref	
Yes	392	185 (47.2)	1.86 (1.51–2.28)		1.48 (1.18–1.84)	0.001
Don’t know	139	31 (22.3)	0.60 (0.40–0.89)	0.01	0.62 (0.41–0.94)	0.02
Missing	9					
**History of past TB**						
No	4,327	1,350 (31.2)	ref		ref	
Yes	810	365 (45.1)	1.81(1.55–2.11)	0.001	1.61 (1.37–1.90)	0.000
Unknown	31					
**Smoke tobacco products**						
No	3,252	1,256 (38.6)	ref		ref	
Yes	1,901	466 (24.5)	0.52 (0.45–0.59)	0.001	0.64 (0.54–0.74)	0.000
Missing	15					
**Consume alcohol**						
No	3,214	1,208 (37.6)	ref		ref	
Yes	1,940	517 (26.7)	0.60 (0.53–0.68)	0.001	0.81 (0.70–0.94)	0.01
Missing	14					

^#^ HIV status was determined by dried blood spot(DBS) test results where this was available and by self-report where DBS was not done. HIV status was unknown where participants declined to disclose status and refused DBS testing

In the regression analysis that excludes participants reporting cough of less than 2 weeks duration, the factors associated care seeking were generally similar to those identified when cough of any duration was used (S4 Table). Older people (aOR 1.61, 95% CI1.37–1.90), those with a history of TB (aOR 1.57, 95% CI 1.32–1.88), and those with diabetes (aOR 1.48, 95% CI 1.18–1.84) were significantly more likely to seek care. Although those living in rural areas were also more likely to seek care than those living in urban areas, this did not reach statistical significance (1.15, 95%CI 1.00–1.32, p = 0.05). Unknown and negative HIV status, smoking tobacco products and consuming alcohol were significant predictors of not seeking care, in this subgroup.

In this survey 234 people had bacteriologically confirmed TB, and among them 98 reported symptoms with 41 having sought care for their symptoms, as previously reported [10]. Among these 98 participants we found no statistically significant differences in healthcare seeking by sociodemographic characteristics and history of TB. Those with unknown HIV status OR 0.16 95% CI 0.03–0.82), p = 0.03, and those who smoked cigarettes (OR 0.39 (0.17–0.89), p = 0.03) were significantly less likely to seek care for their symptoms (Table 3). Among 57/98 (58.2%) participants with bacteriologically confirmed TB who had not sought care for symptoms 38/57 (66.7%) intended to do so, 8/57 (14.0%) regarded their symptoms as benign, and 6/57 (10.5%) reported access barriers (S1 Table).

**Table 3:** Factors associated with self-reported care seeking for TB symptoms in participants with bacteriologically confirmed TB in a community survey, n = 98.

Characteristic	Number reporting symptoms	Sought care	OR 95% CI	p value
**Sex**				
Male	47	19 (40.4)	ref	
Female	51	22 (43.1)	1.12 (0.50–2.50)	0.79
**Age group (years)**				
15–24	8	2 (25.0)	ref	
25–49	50	21 (42.0)	2.17 (0.40–11.84)	0.37
≥50	40	18 (45.0)	2.45 (0.44–13.67)	0.31
**Locality**				
Urban	56	23 (41.2)	ref	
Rural	42	18 (42.9)	1.08 (0.48–2.42)	0.86
^#^ **HIV status**				
HIV positive	31	16 (51.6)	ref	
HIV negative	53	23 (43.4)	0.72 (0.30–1.75)	0.48
HIV status unknown	14	2 (14.3)	0.16 (0.03–0.82)	0.03
**Diabetes(self-report)**				
No	88	38(43.2)	ref	
Yes	7	2 (28.6)	0.53(0.10–2.86)	0.46
Don’t know	3	1 (33.3)	-	
**History of past TB**				
No	71	28 (39.4)	ref	
Yes	27	13 (48.2)	1.43(0.58–3.48)	0.44
**Highest education level achieved**				
None	14	5(35.7)	ref	
Grade 1–12	82	36(43.9)	1.41(0.43–4.57)	0.59
Tertiary	2	0(0)		
**Consume alcohol**				
No	57	28(49.1)	ref	
Yes	41	13 (31.7)	0.48 (0.21–1.11)	0.09
**Smoke tobacco products**				
No	49	26(53.1)	ref	
Yes	49	15 (30.6)	0.39 (0.17–0.89)	0.03

^#^ HIV status was determined by dried blood spot(DBS) test results where this was available and by self-report where DBS was not done. HIV status was unknown where participants declined to disclose status and DBS testing

## Discussion

In this community TB survey, overall, 66.6% of symptomatic participants had not sought care for their symptoms. Among those with symptoms who were diagnosed with bacteriologically confirmed TB more than half had not sought care for their symptoms. The majority of participants delayed seeking care reporting that they were intending to do so. Also concerning is that more than 14% of participants with bacteriologically confirmed TB despite presenting TB suggestive symptoms did not consider their symptoms serious enough to seek care. Timely care seeking and screening for TB enables early detection and initiation of treatment for active TB thus curbing transmission, and also providing opportunity for screening and management of close contacts [11]. Although the four WHO TB screening symptoms have low sensitivity [9], in this survey we previously reported that over 40% of people with bacteriologically confirmed TB reported symptoms(6.8% were symptom screen positive only and 35.0% screen positive on symptoms and CXR) indicating the role of symptoms in finding people with TB [10].

Studies have shown that people with TB symptoms delay seeking care for at least one month [12, 13] which indicates that they contribute to sustained community transmission of TB. In this analysis the survey criteria for symptoms were a duration of at least two weeks for night sweats, weight loss, and fever, and although 60% of participants reported intention to seek care, our findings are nonetheless suggestive of delayed healthcare seeking consistent with the literature [12, 13]. Care seeking was delayed even among those who reported cough of 2 weeks or longer (S2 and S4 Tables). Data from population surveys in South Africa has shown high levels of overall knowledge about TB and its transmission [14, 15] indicating a need to better understand and address the factors that impede prompt action on this knowledge. In this survey many people did not regard TB symptoms as serious enough to seek care. TB symptoms may be mild and may also overlap with those of other respiratory infections, and this finding is not novel. However, given the spectrum of TB disease presentation, propensity for transmission and the need to accelerate action to address the high TB burden in the country, this finding underscores a need to radically change the approach to TB symptoms. The COVID-19 pandemic has elevated the value and feasibility of various models of remote patient interaction and management, with wide use of SMS/WhatsApp messaging for screening and communicating test results. Self-screening on the WhatsApp and SMS-based TB Healthcheck App [16] which aims to increase TB screening and early case detection in South Africa is one such example, which can prompt early action on symptoms. The success of this screening intervention will also depend on its popularization and efficient management of those who self-screen positive and present for additional in-facility screening and testing. Efficient management will include fast tracking of these people to reduce patient waiting times and adherence to infection control policies to manage potential TB transmission in facilities [17].

Other barriers to seeking healthcare for TB symptoms that we found are access barriers that have previously been reported by studies on healthcare access and utilization in South Africa beyond just for TB services, especially in rural areas where distances traveled to health facilities and the related costs are a factor [18, 19]. In this analysis 10% of those with bacteriologically confirmed TB reported access barriers related to distance and travel costs. Interventions such as community-based programmes undertaken by Community Healthcare Workers in the Primary Healthcare model can address some of these distance and travel cost barriers [20]. Community Healthcare Workers also provide opportunity to increase awareness about the significance of and the urgency to act on TB symptoms and to link those at risk to social support programmes that can support people to better access healthcare facilities and throughout the TB diagnosis and treatment journey. People in urban areas likely face other access barriers including the possibility of losing jobs and other opportunities while attending healthcare facilities that may be congested and have long and unpredictable patient wait times [21–23]. In this survey a small proportion of participants reported health system barriers. The 2015 South African Department of Health’s National policy on management of patient waiting times in outpatient departments proposed use of the Central Chronic Medicine Dispensing and Distribution (CCMDD) system, working with Ward-based Primary Healthcare Outreach Teams (WBPHCOTs) that include Community Healthcare Workers in the Primary Healthcare model, appointment systems, SMS/WhatsApp reminders, and patient education to minimize avoidable visits as measures to reduce patient wait times and decongest healthcare facilities [24]. Some of these recommendations including the deployment and work of Community Healthcare Workers in some communities and sending TB test results to patients by SMS have now been implemented [25]. South Africa also has TB workplace polices whose strengthening could support early detection and appropriate management of TB in the workplace [26] and remove delayed care seeking related to fears of job losses and stigma. South Africa is also implementing the Multisectoral Accountability Framework on TB which brings together multiple sectors essential for a successful TB response and this includes other government departments with responsibility for policies such as the TB workplace polices [27].

Access barriers may be exacerbated by stigma, especially given the intertwining of HIV and TB [28, 29], causing people to seek healthcare far from where they live or work. This can exacerbate healthcare seeking costs even though TB services are provided at no cost to patients. Therefore, measures to find people with TB should also address the upstream determinants including stigma and the dual TB and HIV stigma. Participants with TB who had unknown HIV status were less likely to seek care, and unknown HIV status is sometimes linked to fear and stigma that can limit testing for HIV and healthcare seeking for other symptoms [30, 31].

Females, the elderly, and those living in rural areas, were significantly more likely to seek care, whereas those without HIV, those with unknown HIV status and those without previous TB, were less likely to seek care. Men’s lower likelihood to seek care for TB symptoms when compared to women and the possible reasons for this are well documented in the literature [32–34]. Efforts to address these gaps should continue including implementing specific interventions that target men [35] and encouraging men to use self-screening modalities such as the TB Healthcheck App. As expected, participants with the comorbidities of HIV and diabetes investigated in this study were more likely to seek care probably driven by structured appointment-based consultations for these comorbid conditions. In 2020, HIV status was known for 66% of notified TB cases in South Africa and among these 71% were LWH with 89% on ART [6]. Although a proportion of these would have initiated ART at TB diagnosis, with an estimated 62.3% people living with HIV (PLWH) on ART in South Africa 2017 [36], the HIV programme plays a significant role in reaching PLWH who may have TB.

Although, the sample was small, in this analysis, among those diagnosed with TB, unknown HIV status, and cigarette smoking were predictors of delayed healthcare seeking for TB symptoms. Therefore, while focus should remain on people LWH given their significantly higher TB risk, attention is also needed on those without HIV, those with unknown status and those with other risk factors including smoking cigarettes, given conditions that enable TB transmission and acquisition in many communities [6]. Healthcare workers managing other health conditions should also routinely screen their patients for TB.

People who had with TB In the past were more likely to seek care and this was also previously shown in Zambia [37]. This is a notable finding since individuals with a prior history of TB have a greater risk of developing active TB, and their healthcare seeking behavior could reflect better understanding of the significance of TB symptoms. It is important that such individuals receive prompt and appropriate testing and care.

This survey was undertaken before the COVID-19 pandemic which had an impact on healthcare seeking for TB. The COVID-19 pandemic led to reduced TB testing because of various reasons including travel restrictions, restrictions on building occupancy including healthcare facilities, disruption of routine activities, and people’s fear of contracting COVID-19 while attending healthcare facilities [6, 8]. Therefore, healthcare seeking patterns post the peak of the COVID-19 period could be different from those observed at the time of the survey. Desirability bias may also affect the reasons reported for not seeking healthcare in particular since the majority of participants reported that they intended to seek care. We did not collect data on the exact duration of symptoms to determine the extent of delays and further examine the intention to seek care. However, our findings are consistent with the literature indicating that people delay seeking healthcare for TB symptoms [11, 13, 38]. In a Kenyan, survey similar to ours, among survey participants with at least one symptom who did not seek healthcare and provided a reason for this, 82% felt their symptoms were not serious to warrant care [38]. Desirability bias could have also affected reporting of other barriers namely health system barriers and use of other medications. Our data on alcohol intake and cigarette smoking was limited, although our findings are consistent with the literature [39–41]. Given the potential complex and causal pathways and confounding factors from symptoms to care seeking (that we did not investigate), the adjusted odd ratios cannot necessarily be interpreted in the same manner [42].

## Conclusion

People with TB symptoms delayed seeking healthcare for them: many regarded the TB symptoms as benign while others faced travel and cost access barriers. Those with unknown HIV status were significantly less likely to seek care. Strengthening community-based TB awareness and screening programmes together TB self-screening models could increase awareness of the significance of TB symptoms, minimize travel and cost access barriers, and prompt early healthcare seeking and thus enable many people with TB in communities to enter the TB care cascade. These programmes and models should be strengthened together with clear and efficient patient management pathways for those who screen positive and require further testing management in healthcare facilities.

## Supporting information

S1 Table Reasons for not seeking care among participants with symptoms. (DOCX)

S2 Table Reasons for not seeking care for TB symptoms among participants who reported cough of ≥2 weeks with or without any other screening symptoms. (DOCX)

S3 Table Reasons for not seeking care among participants who reported symptoms by symptom type and symptom combinations. (DOCX)

S4 Table Factors associated with self-reported care seeking for TB symptoms among participants who reported cough of ≥2 weeks with or any other screening symptoms in a community survey. (DOCX)

S1 Data Minimum dataset. (XLSX)
