# Supplementary material for: Healthcare seeking patterns for TB symptoms: Findings from the first national TB prevalence survey of South Africa, 2017–2019
Source: PLoS One. 2023 Mar 15;18(3):e0282125. doi: 10.1371/journal.pone.0282125 (PMC10016667; doi:10.1371/journal.pone.0282125)
Supplement: S1 Table — (DOCX) [file pone.0282125.s001.docx]

**Supplementary material: Healthcare seeking patterns for TB symptoms: findings from the first national TB prevalence survey of** **South Africa, 2017-2019**

**Supplementary material, Table 1: Reasons for not seeking care among participants with symptoms**

| **Reason^##^** | **All symptomatic participants N=3,442**  **n (%)** | **Symptomatic participants with bacteriologically confirmed TB N= 57**  **n (%)** |
| --- | --- | --- |
| Still planning to seek care | 2,064(60.0%) | 38(66.7%) |
| Symptoms regarded as not serious | 912(26.5%) | 8(14.0%) |
| Access barriers (cost and distance) | 399(11.6%) | 6(10.5%) |
| Other medication | 36(1.0%) | 1(1.8%) |
| Health system factors | 20( 0.6%) | 1(1.8%) |
| Fear of a TB diagnosis | 4(0.01%) | 0(0.0%) |
| Other | 7(0.2%) | 4(7.0%) |

**^##^**Participants could report more than one reason
