# Supplementary material for: Healthcare seeking patterns for TB symptoms: Findings from the first national TB prevalence survey of South Africa, 2017–2019
Source: PLoS One. 2023 Mar 15;18(3):e0282125. doi: 10.1371/journal.pone.0282125 (PMC10016667; doi:10.1371/journal.pone.0282125)
Supplement: S2 Table — (DOCX) [file pone.0282125.s002.docx]

**Supplementary material, Table 2: Reasons for not seeking care for TB symptoms among participants who reported cough of ≥2 weeks with or without any other screening symptoms, N=2,877**

|  |  | **Reason for not seeking care** | | | |
| --- | --- | --- | --- | --- | --- |
| **Variable** | **Did not seek care**  **n** | **Still planning to seek care**  **n (%)** | **Symptoms regarded as benign n (%)** | **Access barriers**  **n (%)** | **Other reasons** |
| **Sex** |  | p= 0.88 | p=0.07 | p=0.001 |  |
| Male | 1,275 | 758 (59.6) | 359 (28.2) | 125 (9.8) | 33 |
| Female | 1,602 | 957(59.7) | 403 (25.2) | 221 (13.8) | 21 |
| **Age group (years)** |  | p=0.06 | p=0.01 | p=0.001 |  |
| 15-24 | 431 | 245 (56.8) | 137 (31.8) | 35(8.1) | 14 |
| 25-49 | 1,298 | 804(61.9) | 350 (27.0) | 121(9.3) | 23 |
| ≥50 | 1,148 | 666 (58.0) | 275 (24.0) | 190 (16.4) | 17 |
|  |  |  |  |  |  |
| **Locality** |  | p=0.000 | p=0.23 | p=0.000 |  |
| Urban | 1,407 | 911 (64.75) | 387(27.5) | 80(5.7) | 29 |
| Rural | 1,470 | 804 (54.69) | 375(25.5) | 266(18.1) | 25 |
| **Highest**  **education level achieved** |  | p=0.01 | p=0.02 | p=0.000 |  |
| None | 485 | 260(53.61) | 104(21.4) | 119 (24.5) | 2 |
| Grade 1-12 | 2,299 | 1,405(61.1) | 627(27.3) | 223(9.7) | 46 |
| Tertiary | 91 | 50(55.0) | 29(31.9) | 4(4.4) | 8 |
| Missing | 2 |  |  |  |  |
| **HIV status**^#^ |  | p=0.001 | p=0.01 | p=0.01 |  |
| HIV positive | 478 | 299(62.55) | 112 (23.4) | 61(12.8) | 6 |
| HIV negative | 1,816 | 1,113 (61.29) | 469 (25.8) | 196 (10.8) | 38 |
| HIV status unknown | 583 | 303 (51.97) | 181 (31.1) | 89 (15.3) | 10 |
| **Diabetes (self-report)** |  | p=0.91 | p= 0.44 | p=0.17 |  |
| No | 2612 | 1,561 (59.8) | 699 (26.8) | 305 (11.7) | 47 |
| Yes | 173 | 102(59.0) | 41(23.7) | 26 (15.0) | 4 |
| Don’t know | 90 | 52(57.8) | 20(22.2) | 15 (16.7) | 3 |
| Missing | 2 |  |  |  |  |
| **History of past TB** |  | p=0.21 | p=0.02 | p=0.29 |  |
| Yes | 4386 | 242 (62.7) | 83(21.5) | 52(13.5) | 9 |
| No | 2,473 | 1,467(59.3) | 674 (27.3) | 287 (11.6) | 45 |
| unknown | 18 |  |  |  |  |
| **Smoke tobacco products** |  | p=0.02 | p=0.78 | p=0.000 |  |
