# Supplementary material for: Healthcare seeking patterns for TB symptoms: Findings from the first national TB prevalence survey of South Africa, 2017–2019
Source: PLoS One. 2023 Mar 15;18(3):e0282125. doi: 10.1371/journal.pone.0282125 (PMC10016667; doi:10.1371/journal.pone.0282125)
Supplement: S3 Table — (DOCX) [file pone.0282125.s003.docx]

**Supplementary material, Table 3: Reasons for not seeking care among participants who reported symptoms by symptom type and symptom combinations**

|  |  | **Reason for not seeking care** | | | |
| --- | --- | --- | --- | --- | --- |
| **Variable** | **Did not seek care**  **n** | **Still planning to seek care**  **n (%)** | **Symptoms regarded as benign n (%)** | **Access barriers**  **n (%)** | **Other reasons** |
| **Symptoms**  Cough, weight loss, night sweats, fever |  | p=0.001 | p=0.001 | p=0.003 |  |
| 0 0 0 1 | 209 | 118 ( 54.46) | 46 ( 22.01) | 35( 16.75) | 10 |
| 0 0 1 0 | 477 | 265( 55.56) | 141 (29.56) | 47 (9.85) | 24 |
| 0 0 1 1 | 106 | 51(48.11) | 37 (34.91) | 15 (14.15) | 3 |
| 0 1 0 0 | 426 | 228 ( 53. 52) | 137 ( 32.16) | 34 (7.98) | 27 |
| 0 1 0 1 | 29 | 16 (55.17) | 4 (13.79) | 8 (27.59) | 1 |
| 0 1 1 0 | 156 | 75( 48.08) | 53 (33.97) | 21 (13.46) | 149 |
| 0 1 1 1 | 34 | 15(44.12) | 8 ( 23.53) | 6 (17.65) | 5 |
| 1 0 0 0 | 1,001 | 641(64.04) | 232 ( 23.18) | 100 (9.99) | 28 |
| 1 0 0 1 | 275 | 167 (60.73) | 68 ( 24.73) | 20 ( 10.18) | 20 |
| 1 0 1 0 | 210 | 134 ( 63.81) | 45 ( 21.43) | 30 (14.29) | 1 |
| 1 0 1 1 | 148 | 89 (60.14) | 31 ( 20.95) | 22 ( 14.86) | 6 |
| 1 1 0 0 | 96 | 55 ( 57.29) | 28 ( 29.17) | 7 (7.29) | 6 |
| 1 1 0 1 | 41 | 25 (60.98) | 5 ( 12.20) | 6 ( 14.63) | 5 |
| 1 1 1 0 | 80 | 57 (71.25) | 15 (18.75) | 6 ( 7.50) | 2 |
| 1 1 1 1 | 85 | 55(64.71) | 11 (12.94) | 13 (15.29) | 6 |

Symptom: Cough, weight loss, night sweats, fever; 1=symptom reported 0= no symptom reported
