# Supplementary material for: Healthcare seeking patterns for TB symptoms: Findings from the first national TB prevalence survey of South Africa, 2017–2019
Source: PLoS One. 2023 Mar 15;18(3):e0282125. doi: 10.1371/journal.pone.0282125 (PMC10016667; doi:10.1371/journal.pone.0282125)
Supplement: S4 Table — (DOCX) [file pone.0282125.s004.docx]

**Supplementary material, Table 4: Factors associated with self-reported care seeking for TB symptoms among participants who reported cough of ≥2 weeks with or any other screening symptoms in a community survey, N=4,372**

| **Characteristic** | **Number reporting symptoms** | **Sought care n(%)** | **OR (95% CI)** | **p value** | **aOR (95% CI)** | p value |
| --- | --- | --- | --- | --- | --- | --- |
| **Sex** |  |  |  |  |  |  |
| Male | 1,800 | 525 (29.2) | ref |  | ref |  |
| Female | 2,572 | 970 (37.7) | 1.47 (1.29- 1.67) | 0.000 | 1.03(0.88-1.20) | 0.72 |
| **Age group (years)** |  |  |  |  |  |  |
| 15-24 | 528 | 97 (18.4) | ref |  | ref |  |
| 25-49 | 1,813 | 515 (28.4) | 1.76 (1.38- 2.25) | 0.000 | 1.61(1.24-2.08) | <0.001 |
| ≥50 | 2,031 | 883 (43.4) | 3.42 (2.70-4.33) | 0.000 | 3.21 (2.49-4.14) | <0.001 |
| **Locality** |  |  |  |  |  |  |
| Urban | 2,020 | 613 (30.35) | ref |  | ref |  |
| Rural | 2,352 | 1882 (37.5) | 1.38 (1.23-1.55) | 0.000 | 1.15( 1.00-1.32) | 0.05 |
| **Highest education level achieved** |  |  |  |  |  |  |
| None | 856 | 371 (43.34) |  |  | ref |  |
| Grade 1-12 | 3385 | 1086 (32.08) | 0.61 (0.53-0.70 | 0.000 | 0.90 (0.75-1.07) | 0.22 |
| Tertiary | 128 | 37(28.91) | 0.5 2(0.36-0.76) | 0.002 | 1.00 (0.66-1.54 | 0.99 |
| Missing | 3 |  |  |  |  |  |
|  |  |  |  |  |  |  |
| **HIV status**^#^ |  |  |  |  |  |  |
| HIV-positive | 872 | 394(45.18) |  |  | ref |  |
| HIV-negative | 2671 | 855 (32.01) | 0.57(0.49-0.67) | 0.000 | 0.54 (0.45-0.64) | 0.000 |
| HIV status unknown | 829 | 246 (29.67) | 0.51 (0.42-0.63) | 0.000 | 0.41 (0.33-0.51) | 0.000 |
| **Diabetes (self-report)** |  |  |  |  |  |  |
| No | 3912 | 1,300 (33.23) | ref |  | ref |  |
| Yes | 335 | 162 (48.36) | 1.88(1.50- 2.35) | 0.000 | 1.47(1.16-1.87) | 0.002 |
| Don’t know | 117 | 27 (23.08) | 0.60( 0.39-0.93) | 0.02 | 0.66(0.42-0.94) | 0.07 |
| Missing | 8 | 6 |  |  |  |  |
| **History of past TB** |  |  |  |  |  |  |
| No | 3,637 | 1,350 (31.2) | ref |  | ref |  |
| Yes | 708 | 365 (45.1) | 1.77 (1.50- 2.09 | 0.000 | 1.57 (1.32-1.88) | 0.000 |
| Unknown | 27 |  |  |  |  |  |
| **Smoke tobacco products** |  |  |  |  |  |  |
| No | 2750 | 1,256 (38.6) | ref |  | ref |  |
| Yes | 1,609 | 466 (24.5) | 0.49(0.43-0.57) | 0.000 | 0.61 (0.52- 0.73) | 0.000 |
| Missing | 13 |  |  |  |  |  |
| **Consume alcohol** |  |  |  |  |  |  |
| No | 2,714 | 1,208 (37.6) | ref |  | ref |  |
| Yes | 1,646 | 517 (26.7) | 0.59(0.51- 0.67) | 0.000 | 0.80 (0.68-0.93) | 0.01 |
| Missing | 12 |  |  |  |  |  |

^#^ HIV status was determined by dried blood spot(DBS) test results where this was available and by self-report where DBS was not done. HIV status was unknown where participants declined to disclose status and refused DBS testing
